# Size precision in insect eyes

**DOI:** 10.1371/journal.pbio.3002471

**Published:** 2024-01-31

**Authors:** Marco Milán

**Affiliations:** 1 Institute for Research in Biomedicine (IRB Barcelona), The Barcelona Institute of Science and Technology, Barcelona, Spain; 2 Institució Catalana de Recerca i Estudis Avançats (ICREA), Barcelona, Spain

## Abstract

The building of fully functional and well-proportioned individuals relies on the precise regulation of the size of each of their constituting organs. A new study unravels a mechanism that confers precision to size regulation of the adult *Drosophila* eye through morphogen-mediated modulation of cell survival.

In developing multicellular organisms, the final size of each organ is tightly regulated by chemical and mechanical cues. *Drosophila* provides an excellent system in which to genetically identify and molecularly dissect those cues. The exquisite regulation of organ size in this animal is exemplified by the reduced variability observed between the left and right wings of the same insect (fluctuating asymmetry, FA [1–4]). A new study unravels an organ-intrinsic mechanism of growth control in the developing fly eye that confers size precision through feedback interactions between proliferating and differentiating cells [5]. This mechanism reduces eye size variability between and within animals, thus contributing to the symmetry between contralateral eyes and having a clear potential impact on eye functionality. In the growing eye primordium, a wave of differentiation moves anteriorly, whereby proliferative progenitors located anterior to the wave are recruited as differentiating retinal cells that exit the cell cycle (Fig 1). When the wave reaches the anterior-most region of the primordium, no remaining progenitors remain in the tissue, and the final eye size is attained. The movement of the differentiation wave relies on the activity of 2 morphogens, the BMP homolog Dpp and Hedgehog (Hh), which are produced by differentiating retinal cells that signal anteriorly to nearby proliferating cells to recruit them as new differentiating retinal cells. In this regard, Casares, Ares, and colleagues reveal the role of Dpp in regulating eye size by blocking the apoptosis of progenitor cells and present evidence that this mechanism plays a key role in reducing eye size variability and FA.

**Fig 1:**
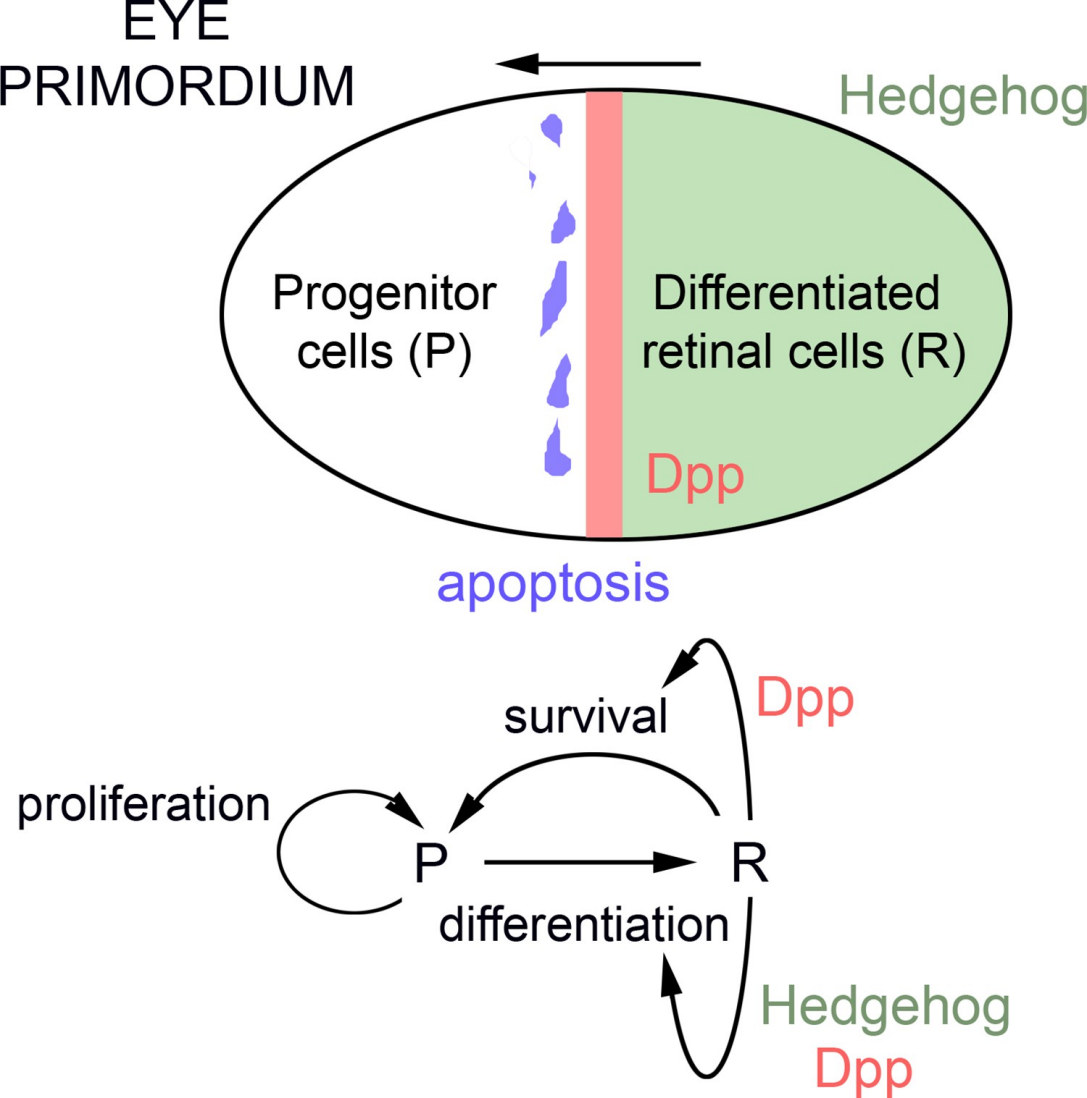
Cartoons depicting the development of the *Drosophila* eye primordium and the roles of Dpp and Hedgehog morphogens in regulating differentiation and survival. A wave of differentiaton—which relies on the activity of 2 morphogens, Dpp (in pink) and Hedgehog (in green)—moves anteriorly (see arrow). Proliferative progenitors (P) located anterior to the wave get recruited as differentiating retinal cells (D) that exit the cell cycle. Dpp provides a survival cue in P cells by repressing cell death (in purple).

Cell death is involved in the removal of excess cells in the differentiating side of the eye primordium [6]. Using a sensitive antibody to detect apoptotic cells in the developing eye primordium, Casares, Ares, and colleagues detected the presence of apoptotic cells also in the progenitor cell population and present evidence that these cells are distributed in a stripe abutting the differentiation wave ([5], Fig 1). When apoptosis was blocked in this stripe by targeted expression of a transgene carrying synthetic miRNAs against the pro-apoptotic genes *hid*, *reaper*, and *grim*, the resulting eyes became significantly larger. Most interestingly, eye size dispersion and FA were both enhanced under these experimental conditions, thereby pointing to a role of apoptosis not only in regulating eye size but also in reducing size variability. In the search for the signal responsible for regulating cell death and survival in the progenitor cell population abutting the differentiation wave, the authors centered their attention on the potential implication of Dpp, given its transient expression in newly differentiated retinal cells. The depletion of the Dpp receptor in progenitor cells caused a dramatic increase in apoptotic rates with a concomitant reduction in eye size. Also under these conditions, size variability and FA increased markedly. The impact of Dpp receptor depletion on eye size and size variability was rescued upon intracellular derepression of the Dpp pathway or apoptosis blockage. In flies carrying the *Bar* mutation, *dpp* expression in newly differentiating retinal cells is partially compromised [7] and the resulting adult eyes are smaller, more variable in size, and highly asymmetric [5]. Intracellular derepression of the Dpp pathway or apoptosis blockage rescued eye size, variability, and asymmetry in *Bar* mutant flies. All these experimental observations led to 2 major conclusions. First, differentiating retinal cells modulate the size of the adult eye by regulating the number of progenitor cells ready to be recruited as retinal cells. Mechanistically, differentiating retinal cells exert this activity by promoting the survival of progenitor cells through Dpp. Second, any discrepancy in the sizes of the differentiated and progenitor cell populations will be adjusted through modulation of the propensity of progenitor cells to die. In other words, the susceptibility of the progenitor cell population to enter apoptosis contributes to reducing size variability and symmetry, thereby suggesting that this feedback mechanism contributes to the precision of eye size. To test this notion, the authors built and analyzed a minimalistic mathematical model of the process that recapitulated the dynamics of eye differentiation and the acquisition of final eye size and the precision of this process (Fig 1). After fitting it to an experimentally determined growth curve of the eye primordium [8], this model supported the notion that Dpp regulates stability through apoptosis and that the high propensity of apoptosis in progenitor cells is indeed required for robust size regulation. To experimentally test whether this newly identified mechanism can accommodate changes in final organ size while maintaining precision, the authors overloaded the growing primordium with high levels of the mitogenic molecule Upd, which activates the JAK/STAT pathway and regulates eye growth [8]. Under these conditions, the eyes became larger but remained highly symmetric. These results challenge Waddington’s proposal that all mutant conditions cause developmental instability, and they reinforce the reported roles of Dpp and apoptosis not only in regulating tissue size but, most importantly, in conferring size precision. Apoptosis has previously been shown to contribute to reducing size variability and FA in adult wings [9,10]. Thus, apoptosis appears to impact the ability of flies to be the queens of the air through precise regulation of the size of their left and right eyes and wings.
